# Ocular surface assessment and morphological alterations in meibomian glands with non-contact meibography in electronic cigarette smokers

**DOI:** 10.5935/0004-2749.20230069

**Published:** 2023

**Authors:** Mustafa Kalayci, Ersan Cetinkaya, Lütfiye Yaprak, Kenan Yigit, Elcin Suren, Berna Dogan, Muhammet Kazim Erol

**Affiliations:** 1 Department of Ophthalmology, Antalya Training and Research Hospital, University of Health Sciences, Antalya, Turkey

**Keywords:** Electronic nicotine delivery systems, Meibomian glands, Meibomian gland dysfunction/diagnosis, Smoking/adverse effects, Vaping/adverse effects, Diagnostic techniques, ophthalmological, Sistemas eletrônicos de liberação de nicotina, Glândulas tarsais, Disfunção da glândula tarsal/diagnostico, Fumar/efeitos adversos, Vaping/efeitos adversos, Técnicas de diagnóstico oftalmológico

## Abstract

**Purpose:**

The study aimed to evaluate the ocular surface and meibomian gland morphology in electronic cigarette (e-cigarette) smokers.

**Methods:**

The upper and lower eyelids of 25 male e-cigarette smokers and 25 healthy male non-smoker patients were evaluated using Sirius meibography. Meibomian glands loss was automatically calculated using Phoenix meibography imaging software module, with the result obtained as percentage loss. Ocular Surface Disease Index (OSDI) questionnaire, tear breakup time test, and Schirmer II test were administered and performed in all cases.

**Results:**

The mean e-cigarette smoking duration was 4.9 ± 0.9 (range, 3.4-7) years. While the mean Schirmer II test value was 9.16 ± 2.09 mm in e-cigarette group, it was 11.20 ± 2.14 mm in control group (p=0.003). Mean tear breakup time was 6.96 ± 2.31 seconds in e-cigarette group and 9.84 ± 2.13 seconds in control group (p=0.002). The mean OSDI value was 28.60 ± 6.54 and 15.16 ± 7.23 in e-cigarette and control groups, respectively (p<0.001). In Sirius meibography, the average loss for the upper eyelid was 23.08% ± 6.55% in e-cigarette group and 17.60% ± 4.94% in control group (p=0.002), and the average loss for the lower eyelid was 27.84% ± 5.98% and 18.44% ± 5.91%, respectively (p<0.001). Additionally, a significant positive correlation was identified between the loss rates for both upper and lower eyelid meibography with e-cigarette smoking duration (r=0.348, p<0.013 and r=0.550, p<0.001, respectively).

**Conclusion:**

Long-term e-cigarette smoking causes damage to the meibomian glands; therefore, meibomian gland damage should be considered in ocular surface disorders due to e-ci­garette smoking.

## Abstract (pt)

**Objetivo:**

Avaliar a superfície ocular e a morfologia da glândula meibomiana em usuários de cigarros eletrônicos.

**Métodos:**

Foram avaliadas através de meibografia Sirius as pálpebras superiores e inferiores de 25 usuários de cigarros eletrônicos do sexo masculino e 25 pacientes não usuários saudáveis, também do sexo masculino. A perda nas glândulas meibomianas foi calculada automaticamente com o módulo de software de imagem de meibografia Phoenix. O resultado foi obtido como perda percentual. O questionário *Ocular Surface Disease Index* (OSDI), o teste do tempo de ruptura lacrimal e o teste de Schirmer II foram administrados em todos os casos.

**Resultados:**

A duração média do uso de cigarros eletrônicos foi de 4,9 ± 0,9 anos (intervalo de 3,4-7 anos). O valor médio do teste de Schirmer II foi de 9,16 ± 2,09 mm no grupo de usuários de cigarros eletrônicos e de 11,20 ± 2,14 mm no grupo controle (p=0,003). O valor médio do teste do tempo de ruptura lacrimal foi de 6,96 ± 2,31 segundos no grupo de usuários de cigarros eletrônicos e 9,84 ± 2,13 segundos no grupo controle (p=0,002). O valor médio do *Ocular Surface Disease Index* foi de 28,60 ± 6,54 e 15,16 ± 7,23 para os grupos de usuários de cigarros eletrônicos e controle, respectivamente (p<0,001). Na meibografia de Sirius, a perda média para a pálpebra superior foi de 23,08 ± 6,55% para o grupo de usuários de cigarros eletrônicos e 17,60 ± 4,94% para o grupo controle (p=0,002), e a perda média para a pálpebra inferior foi de 27,84 ± 5,98% e 18,44 ± 5,91%, respectivamente (p<0,001). Além disso, foi observada uma correlação positiva significativa entre a taxa de perda na meibografia palpebral superior e inferior com a duração do tabagismo eletrônico, respectivamente de (r=0,348, p<0,013) e (r=0,550, p<0,001).

**Conclusão:**

O uso prolongado de cigarros eletrônicos causa danos às glândulas meibomianas. Portanto, esses danos devem ser considerados em distúrbios da superfície ocular devidos ao uso desses dispositivos.

## INTRODUCTION

Electronic cigarettes (e-cigarettes) are becoming increasingly popular^(1)^; however, there are still differences in health effects perception of e-cigarettes in society. Having no tobacco content and being perceived as less harmful for health are among the main reasons for the significant increase in e-cigarette smoking, especially among young people^(2)^. However, although e-cigarettes do not contain tobacco, they include vegetable glycerin and propylene glycol, flavors, and a liquid often made from nicotine. The e-cigarette liquid is electrically heated to generate vapor for inhalation. Therefore, the mixture is turned into an aerosol, and the vapor is inhaled by the smoker^(3)^. However, e-cigarettes also contain volatile organic compounds, such as formaldehyde and acetaldehyde, heavy metals, including chromium, nickel, and cadmium, polycyclic aromatic hydrocarbons, and other harmful substances, e.g., nitrosamine and cotinine, which are nicotine derivatives^(2)^.

Meibomian glands are sebaceous glands located in the tarsi on the lower and upper eyelids, although being more abundant in the upper eyelid^(4)^. Meibomian gland dysfunction is a chronic abnormality characterized by terminal duct obstruction or structural and functional changes in glandular secretion^(5)^. Meibomian gland dysfunction is the most important cause of evaporation-associated dry eyes and causes instability in the tear film due to lipid layer deficiency^(6)^. This dysfunction can be analyzed by indirect methods, such as tear breakup time (TBUT) and Schirmer tests, or direct techniques, such as meibography. While the results of indirect tests may be interpreted differently by each practitioner, meibography provides detailed objective anatomical meibomian gland evaluation^(7)^.

In the literature, there are two studies investigating the effects of e-cigarettes on ocular health. Munsamy et al. evaluated corneal thickness and TBUT before and after e-cigarette liquid inhalation^(8)^. Isa et al. investigated tear function in e-cigarette smokers using TBUT, tear meniscus height, Schirmer test, and Ocular Surface Disease Index (OSDI)^(9)^. However, to the best of our knowledge, there has been no research demonstrating the direct effect of e-cigarette smoking on meibomian glands.

This study aimed to determine the structural damage that may be caused by e-cigarette smoking in meibomian glands using Sirius meibography and evaluate its correlation with indirect methods, including OSDI assessment, TBUT test, and Schirmer II test.

## METHODS

This prospective, cross-sectional study included 25 male e-cigarette smokers and 25 healthy male non-smokers. Control group comprised healthy volunteers who applied for a routine eye examination in our clinic. This group included healthy non-smokers without a smoking history and smokers in their close family. In this group, those who had refractive surgery, dry eye disease, or used contact lenses or artificial tear drops due to dry eye disease were excluded from the study. Additionally, current smokers, former smokers (who had quit smoking less than 1 year before the study), those with active disease or infection, and those with additional systemic disease (diabetes mellitus, hypertension, etc.) were excluded from the study.

Control group participants were asked whether they were exposed to e-cigarette smoking at home or work to exclude passive e-cigarette smokers. Volunteers exposed to e-cigarette smoke for more than 30 minutes daily were considered passive smokers and excluded from the study. All volunteers underwent a complete eye examination, including fundoscopy. Participants’ left eyes were evaluated during the examinations and measurements. The study protocol was approved by the Ethics Committee of Antalya Training and Research Hospital. All clinical procedures were performed according to the principles of Declaration of Helsinki. Written informed consent was obtained from all participants.

Patients with a history of ocular surgery, any ocular or systemic disease, any chronic drug use (antiandrogens, antidepressants, antihistamines, or drugs that may cause meibomian gland dysfunction, such as isotretinoin) and those with regular alcohol consumption (consuming more than 4 drinks on any day or more than 14 drinks weekly) were excluded from the study. The inclusion criteria for e-cigarette group were using e-cigarettes regularly for at least three years, smoking e-cigarettes with their containing at least 50% propylene glycol and at least 3 mg/ml nicotine, vaporing at least 3 ml e-cigarette liquid per day, and having a best-corrected visual acuity of at least 20/20.

An OSDI questionnaire administration, TBUT test, Schirmer II test, and Sirius meibography were consecutively performed by an experienced ophthalmologist blinded to the participants’ group (single-blinded). A detailed ophthalmological examination, including best-corrected visual acuity, intraocular pressure, slit-lamp biomicroscopy, and dilated fundoscopy were performed in all patients. Then, participants’ symptoms were evaluated with OSDI (Allergan, Irvine, CA, USA) questionnaire, which is a 12-item questionnaire evaluating ocular irritation symptoms due to dry eye and patients’ visual function. The questionnaire items are related to ocular symptoms, environmental stimuli, and visual function. The respondent marked the severity of being affected by each symptom on a scale from 0 (never) to 4 (always)^(10)^.

For TBUT evaluation, a participant was placed in front of a biomicroscope, sodium fluorescein strips were inserted into the lower fornix for a short time, and the cornea was stained through blinking. During the biomicroscopic examination, the participant was asked not to blink while a cobalt blue light shined into the eye, and the time when the first black spots or lines formed in the corneal tear layer was recorded in seconds. This procedure was repeated three times, and the average of three measurements was determined as TBUT value. TBUT of less than 10 seconds was considered abnormal.

For Schirmer II test, a local anesthetic drop was instilled into the participant’s eye, and Schirmer paper of 5-mm width and 35-mm length with a bent end was inserted into the one-third lateral part of the lower lid and removed after 5 minutes. The wetted length from the bent end was measured and recorded in mm. A tear secretion value of less than 5 mm was considered abnormal.

**Figure 1:**
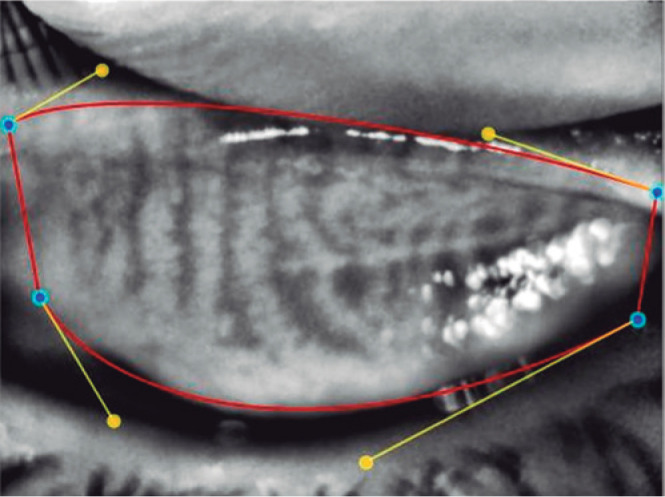
The eyelids borders were marked using the Phoenix software module.

**Figure 2:**
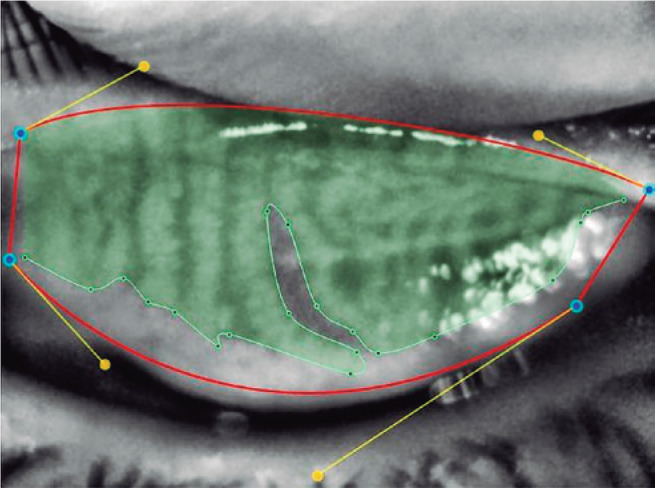
The meibomian glands were marked using the Phoenix software module.

**Figure 3:**
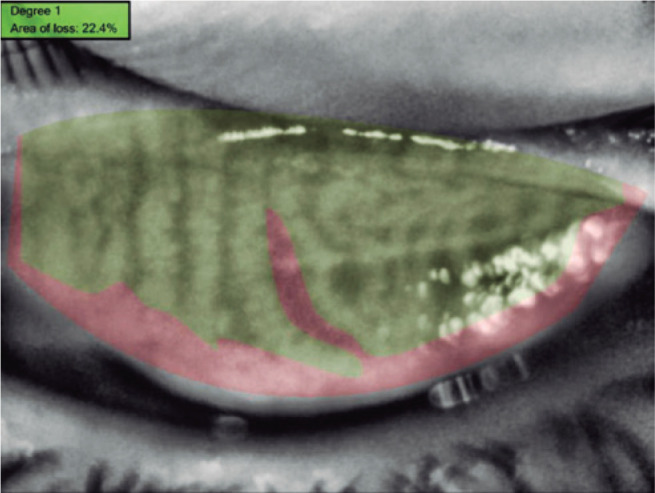
The loss in the meibomian glands was automatically calculated using the Phoenix software module.

Finally, non-contact meibography was applied to participants’ left eye. This procedure was performed using infrared light with Phoenix meibography imaging software module installed on Sirius (CSO, Florence, Italy) corneal topography device. The participant’s head was placed on the device, and they were asked to look straight ahead. First, the lower eyelid was inverted by pressing on its outer part, and meibomian glands measurements were taken from the tarsal conjunctival surface of the lower eyelid. The same procedure was repeated for the upper eyelid. At least three measurements were made, and the best image obtained was evaluated. During the image analysis, the borders of the eyelids and then those of the meibomian glands were marked using the Phoenix software module (Figure 1 and Figure 2). The meibomian glands loss was automatically calculated using the same software. The result was obtained as percentage loss (Figure 3).

### Statistical analysis

The data obtained were recorded using SPSS version 23.0 (IBM, Armonk, NY, USA). Descriptive statistical methods (mean and standard deviation) were used to evaluate the data. Data distribution was analyzed using Shapiro-Wilk test, and the parameters were distributed normally. Independent t-test was applied to all parameters. Pearson correlation test was conducted to examine the relationships between variables. Data evaluations were undertaken at the 95% confidence interval, and p values of less than 0.05 were considered statistically significant.

## RESULTS

Twenty-five e-cigarette smokers and 25 healthy non-smoker controls were included in the study. All participants in both groups were male. The mean age was 28.8 ± 3.1 (range, 23-35) years in e-cigarette group and 28.6 ± 3.4 (range, 22-35) years in control group (p=0.680). The mean e-cigarette smoking duration was 4.9 ± 0.9 (range, 3.4-7) years. The mean Schirmer II test value was 9.16 ± 2.09 mm in e-cigarette group and 11.20 ± 2.14 mm in control group (p=0.003). The mean TBUT test value was 6.96 ± 2.31 seconds in e-cigarette group and 9.84 ± 2.13 seconds in control group (p=0.002). The mean OSDI score was 28.60 ± 6.54 in e-cigarette group and 15.16 ± 7.23 in control group (p<0.001) (Table 1).

**Table 1:** Participants’ tear function test results and percentage of loss in Sirius meibography

	E-cigarette group	Control group	p-value
Schirmer II (mm/5 min)	9.16 ± 2.09	11.20 ± 2.14	**0.003**
TBUT (s)	6.96 ± 2.31	9.84 ± 2.13	**0.002**
OSDI	28.60 ± 6.54	15.16 ± 7.23	**<0.001**
Loss on upper eyelid meibography (%)	23.08 ± 6.55	17.60 ± 4.94	**0.002**
Loss on lower eyelid meibography (%)	27.84 ± 5.98	18.44 ± 5.91	**<0.001**

TBUT= tear breakup time; OSDI= Ocular Surface Disease Index.

An average loss in the upper eyelid Sirius meibography was 23.08% ± 6.55% in e-cigarette group and 17.60% ± 4.94% in control group (p=0.002). According to the same method, the average loss in the lower eyelid was 27.84% ± 5.98% in e-cigarette group and 18.44% ± 5.91% in control group (p<0.001) (Table 1).

A significant correlation was found between the upper and lower eyelid meibography loss rates and the OSDI score. In addition, a significant positive correlation was identified between the loss rates of both upper and lower eyelid meibography and e-cigarette smoking duration (Table 2).

**Table 2:** Correlation of loss rates on Sirius meibography with OSDI, TBUT, and Schirmer II results and e-cigarette smoking duration

	OSDI	TBUT	Schirmer II	E-cigarette smoking duration
Loss on upper eyelid meibography	**r=0.300** p=0.034	r=-0.084 p=0.560	r=-0.204 p=0.155	**r=0.348** p=0.013
Loss on lower eyelid meibography	**r=0.494** p<0.001	r=-0.136 p=0.348	r=-0.242 p=0.091	**r=0.550** p<0.001

TBUT= tear breakup time; OSDI= Ocular Surface Disease Index.

**Figure 4:**
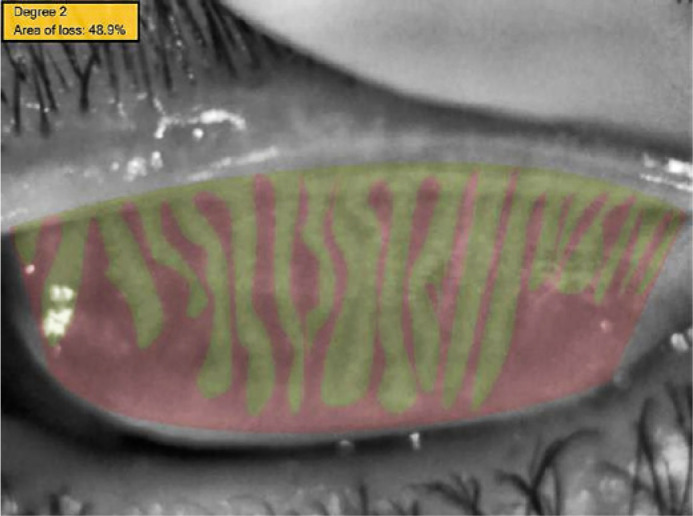
The meibomian gland loss rate of a patient’s upper eyelid from study group.

In addition to these findings, in Sirius meibography, the meibomian glands were unevenly distributed, thinner, and less hyperreflective, the gland folds and the distance between the glands increased, and the glands did not extend to the orifices among the participants that smoked e-cigarettes (Figure 4). In contrast, in control group, the meibomian glands were regular and thick, had high hyperreflectivity, and extended to the orifices.

## DISCUSSION

In this study, the chronic effects of e-cigarette smoking on the meibomian glands in otherwise healthy men were investigated. E-cigarette smokers had significant meibomian glands loss compared to the controls. In addition, as e-cigarette smoking duration increased, meibomian gland damage also increased.

The changes in meibomian gland morphology in e-ci­garette smokers have not yet been investigated. To our knowledge, this is the first study to objectively investigate the anatomical and morphological changes in the meibomian glands using non-contact meibography in e-cigarette smokers.

The literature contains studies evaluating the effects of tobacco-containing cigarettes on meibomian glands^(11-13)^. Muhafiz et al.^(11)^ investigated the effects of tobacco-containing cigarettes on meibomian glands using meibography, showing a significant loss in the meibomian glands of the upper eyelid in study group compared to control group. However, they did not find a significant meibomian gland loss in the lower eyelid meibography. The authors attributed this difference to lower eyelid eversion difficulty in meibography. They also suggested that as airborne smoke particles moved against gravity, they had a greater effect on the meibomian glands in the upper eyelid. In contrast, Gulmez Sevim et al.^(14)^ demonstrated that Sirius meibography was a reliable method to evaluate the meibomian gland in both lower and upper eyelids. In our study, the significant loss in meibomian glands in both lower and upper eyelids in e-cigarette smokers and the meibomian gland loss being greater in the lower eyelid compared to the upper eyelid suggest that the effects of e-cigarette smoking might be different compared to tobacco-containing cigarette smoking. This difference can be explained by the composition of tobacco cigarettes, which contain more than 4,000 additives, whereas the e-cigarette liquid comprises fewer elements, although they are more intense in content. The main components of e-cigarette liquid are nicotine, propylene glycol, vegetable glycerin, flavoring, and distilled water. When e-cigarette liquid is heated to a certain temperature by the device for vaporization, propylene glycol and vegetable glycerin produce oxidative stress factors, such as free radicals and volatile carbonyls, through thermal degradation^(15)^. Additionally, overheated propylene glycol can turn into formaldehyde^(16)^. In their recent study, Vitoux et al.^(17)^ have demonstrated that exposure to formaldehyde gas increased inflammation *in vitro* dry eye model. The researchers used conjunctival cells to show that formaldehyde exacerbated cell death and inflammation, and found increased interleukin *(IL)-6* and *IL-8* gene expression after a 15-minute exposure. In another study evaluating the effect of formaldehyde on the ocular surface, Salem et al.^(18)^ assessed the ocular surface of rats exposed to gaseous formaldehyde released from a piece of cotton soaked with 10% formaldehyde and placed 15 cm away from the rats for two hours a day for five days a week over a two-week period. This *in vivo* model revealed significant epithelial cell necrosis and corneal damage^(18)^. When the results of both studies are evaluated together with our findings, formaldehyde can be considered to have toxic effects on the meibomian glands, thereby causing damage to the gland cells. Furthermore, it can be suggested that the toxic effect of formaldehyde on meibomian glands is an important contribution to ocular surface instability.

While oxidative stress may be caused by reactive oxygen species production by inflammatory cells, it can also trigger the formation of ocular surface inflammation. Oxidative free radicals directly oxidize various macromolecules, including lipids. More than 200 types of aldehydes, including malondialdehyde, arise from lipid oxidative degradation in the cellular membrane, also known as lipid peroxidation. In the literature, lipid peroxides and their degradation products have been described to directly or indirectly affect many functions integrated into cellular and organ homeostasis^(19,20)^. In our study, we showed that e-cigarette smoking increased OSDI score and decreased TBUT and Schirmer II test results. The overexpression of reactive oxygen species production in the ocular surface may be triggered by prolonged exposure to atmospheric oxygen and insufficient antioxidant support due to tear film imbalance^(21)^. Menicagli et al.^(22)^ compared malondialdehyde levels in e-cigarette and tobacco smokers and a control group. They showed that the malondialdehyde levels in e-cigarette and tobacco smokers were higher compared to the controls, but those in e-cigarette and tobacco smokers were similar^(22)^. Choi et al.^(23)^ reported that late lipid peroxidation markers and malondialdehyde expression were increased in the tear film and ocular surface in patients with dry eyes. Malondialdehyde levels were associated with TBUT, Schirmer score, conjunctival goblet cell density, and symptom score^(23)^. In another study, Wakamatsu et al.^(24)^ determined that reactive oxygen species production was associated with cell membrane lipid peroxidation and inflammatory cell infiltration in the ocular surface-lacrimal gland unit. Therefore, it can be considered that the decrease in the dry eye test values (TBUT and Schirmer test) and increase in the symptom score (OSDI assessment) among e-cigarette smokers may be due to the direct effects of lipid peroxidation products, such as malondialdehyde. In addition, in our study, the significant correlation between the upper and lower eyelid loss rates in meibography and the OSDI score suggested that lipid peroxidation products might also have an indirect effect on the symptom score by damaging meibomian glands. Moreover, according to our findings, the impairment in the dry eye tests was not correlated with the loss rate in upper and lower eyelid meibography, but the correlation of the symptom score with the loss rate in upper and lower eyelid meibography was significant, suggesting that meibomian gland dysfunction affected symptom score (OSDI assessment) earlier than dry eye tests (Schirmer test and TBUT).

E-cigarette batteries contain lithium, manganese, and cadmium, and heating wires comprise a combination of aluminum, chrome, lead, nickel, and some other metals. The main reason for the detection of many toxic metals in e-cigarette liquid and aerosol is the mixing of toxic metals into the liquid from the heated wires and batteries^(25)^. In a comprehensive study conducted in Taiwan, Lian et al.^(26)^ reported that the incidence of sicca syndrome was 3.6 times higher in areas with farm soil high in chromium and nickel. In addition, the same researchers exposed the salivary glands of mice to different heavy metals to directly evaluate their effects on salivary glands and examined the epithelial cells of these glands. They revealed that chromium, in particular, triggered salivary gland cells death. Considering that the lacrimal gland structure is similar to that of the salivary gland and both glands are commonly affected through the same inflammatory process in diseases, such as Sjörgen syndrome, heavy metals contained in e-cigarettes may have a toxic effect on the lacrimal glands in e-cigarette smokers. In our study, lacrimal gland dysfunction might have played a role in both the increase in the OSDI score and deterioration in dry eye test results. This effect might have also been enhanced by heavy metals accumulation in the aerosol originating from e-cigarette vapor by contact with the lacrimal glands and heavy metals mixed into the blood from the lungs (dual effect). Moreover, heavy metals may accumulate in meibomian glands through this dual mechanism and contribute to the morphological changes in these glands, which we measured by meibography. Further studies examining lacrimal gland and meibomian gland biopsies in e-cigarette smokers are needed to obtain clearer data on this subject.

The limitation of our study was a relatively small sample size. Another limitation was that our study was cross-sectional. Cross-sectional research does not help determine the cause-and-effect relationship and is susceptible to bias due to low response and misclassification due to recall bias. Accordingly, the variability in e-cigarette use duration and frequency among participants may not fully explain the change in e-cigarette exposure of meibomian glands. The strength of our study was that it was the first to evaluate the meibomian glands anatomically in e-cigarette smokers.

In conclusion, although the exact mechanism underlying the effect of e-cigarette smoking on meibomian gland dysfunction is yet unknown, and perhaps more than one mechanism is involved, we consider that an increased inflammatory reaction induced by e-cigarette smoking may play a role in causing meibomian gland dysfunction. The findings obtained in our study suggest that chronic e-cigarette smoking may be associated with meibomian gland dysfunction and dry eyes.
